# Biopsychosocial Approach for Conservative Management of Abdominal Pregnancy in Previously Infertile Mother

**DOI:** 10.1155/2022/3021097

**Published:** 2022-08-30

**Authors:** Junita Indarti, Yudianto Budi Saroyo, Beta Andewi Resti Anggraheni, Andika Widyatama

**Affiliations:** Department of Obstetrics and Gynecology, Faculty of Medicine, Universitas Indonesia, Jakarta, Indonesia

## Abstract

**Background:**

Abdominal pregnancy is a rare condition in which early termination is generally recommended. However, there are cases of abdominal pregnancies treated using expectant management with satisfactory outcomes. This management may be considered in special cases, such as previously infertile couples.

**Case:**

A case of a 27-year-old woman with infertility history complaining of vaginal bleeding was presented. Physical and ultrasound examination revealed an abdominal pregnancy with 18 weeks of gestation. Although she was ready to abort the pregnancy, she insisted to try expectant management for her pregnancy. Her baby was finally born at 25 weeks via laparotomy.

**Conclusion:**

Abdominal pregnancy is a rare and highly morbid form of ectopic pregnancy. It demands a skilled approach in management. A comprehensive and holistic approach is required to deliver the best outcome for the patient and her family.

## 1. Introduction

Infertility is a common clinical problem affecting 10–15% of all couples worldwide [1]. Infertility is not only a medical problem but also is considered a social problem. There are many impacts of infertility on a couple and their environment. The feeling of depression, grief, guilt, and shame may become an additional problem, along with social isolation experienced by those suffering from infertility [1].

Abdominal pregnancy is a form of ectopic pregnancy of which the sites of implantation included Douglas pouch, omentum, pelvic sidewall, bowel, liver, diaphragm, and large pelvic vessels [2]. The abdominal pregnancy rate ranges between 1 : 10,000 and 1 : 30,000 in the population, representing 1% of all extrauterine pregnancies [3]. Main risk factors for abdominal pregnancy include tubal damage, pelvic inflammatory disease, endometriosis, assisted reproduction techniques, and multiparity [4].

Abdominal pregnancy is rather difficult to be distinguished from a tubal pregnancy or even a normal pregnancy. Early surgical termination is generally recommended to avoid further maternal risks. However, there are also cases of live birth following conservative management of abdominal pregnancy [5]. It is deemed as highly morbid and usually reserved for special populations, one of which is couples with the history of infertility.

This study aimed to present a case of a biopsychosocial approach for conservative management of abdominal pregnancy in previously infertile couple.

## 2. Case

A 27-year-old G1 woman with 18^th^ weeks of gestation came to our obstetrics and gynecology policlinic with chief complaint of abnormal vaginal discharge since a day prior. There was no complaint of vaginal bleeding, urinary problem, nor defecation problem. Previously, she had her routine antenatal care at an obstetrician located in another city. Previously, she thought she had normal pregnancy. However, it was known that her pregnancy was an abdominal pregnancy since 3 weeks ago (15^th^ weeks of gestation). Afterward, she was referred to other hospital in our city. She then routinely took her antenatal examination every 2 weeks.

Previously, her husband and she were suffering from 3 years of primary infertility. She had never used any kind of contraceptive. Her prior medical condition and her family history were also unremarkable.

The patient's vital sign was stable at the time of arrival. Physical examination revealed her fundal height matched the gestational age. Her fetal heart rate was 145 times per minute, while her vaginal discharge was identified as flour albus, suspected to be caused by bacterial vaginosis. Her laboratory results revealed mild microcytic hypochromic anemia and mild hypokalemia.

**Figure 1:**
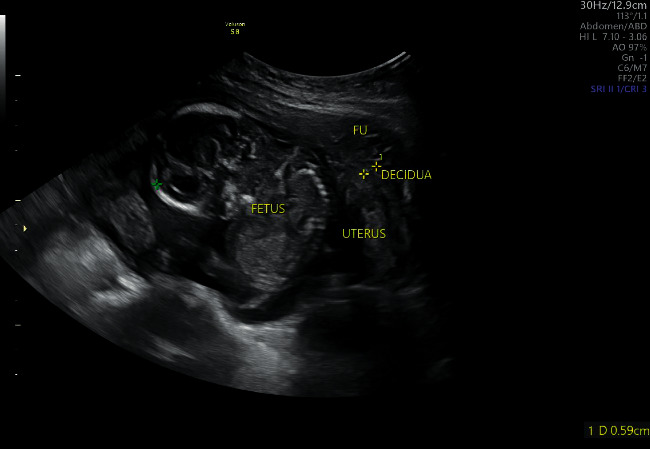
Ultrasonographic image of the abdominal pregnancy.

Ultrasonographic examination revealed singleton abdominal pregnancy with normal biometry and normal activity. The gestational sac was located in the pouch of Douglas, while the placenta was implanted at the omentum. There was no congenital anomaly identified on the fetus (Figure 1).

A family meeting consisting of the patient, her husband, and the obstetricians was commenced following the examination. She was told that abdominal pregnancy was a highly morbid form of ectopic pregnancy; thus, there were significant risks associated with it. However, she was also told that there might be hope for satisfactory maternal and fetal outcomes. She told the obstetricians that her husband and she were suffering badly due to her previous infertility. They also experienced being gossiped by their families and neighbors. Therefore, she hoped that she can retain her pregnancy as they were unsure if she could have any other pregnancy. Eventually, the meeting ended with agreement of trying expectant approach for the pregnancy.

She was a housewife, while her husband was a merchant at a traditional market. Although they are suffering from their previous infertility, they could not afford to seek out medical help due to financial problem. Moreover, there was no sufficient medical infrastructure to address their problem in their city. On the third day following the meeting, there was no complaint remained. She was then discharged and planned to have antenatal care every 2 weeks at our center.

She had her antenatal care on our center. There was no sign of complication on the examination. Ultrasonographic exam showed estimated fetal weight of 297 g and placenta was implanted to omentum. It corresponded to 19 weeks of gestational age. During US Doppler examination, there was a absent end diastolic in the umbilical artery. Based on the data, additional heparin was initiated.

During her 25^th^ gestational week, reverse A wave ductus venosus with brain sparing effect and reverse end diastolic umbilical artery was found. It was decided to deliver the fetus via laparotomy. Due to placenta implantation on the mesenteries, the laparotomy was done with assistance of digestive surgeons.

**Figure 2:**
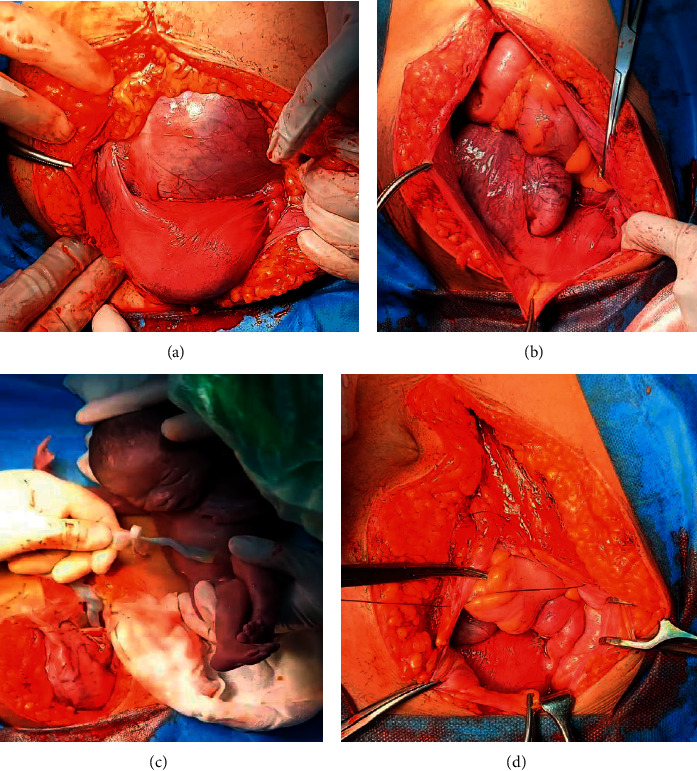
(a) Abdominal gestational sac. (b) The placenta. (c) Delivering the baby. (d) The placenta left in site.

During the surgery, abdominal gestational sac was seen, covered by the right broad ligament, behind the uterus, filling the Douglas cavity. The uterus was slightly enlarged in front of the abdominal gestational sac. After delivering the baby, the placenta was seen to be implanted on the lateral side of the posterior uterine. Thus, placenta was left in site in order to prevent massive hemorrhage. The abdomen was then closed (Figure 2).

The baby was sent to neonatal intensive care unit for further evaluation and treatment. Meanwhile, the mother was observed for 24 hours at intensive care unit and discharged two days following the surgery. Two days later, the baby died due to respiratory distress.

A year after the surgery, the mother was alive and well. Upon follow-up examination, the placenta left was already reduced in size, while the mother had started another pregnancy program.

## 3. Discussion

### 3.1. Biological Aspect

Infertility is a common problem affecting up to 15% of all couples worldwide [1]. It was categorized into primary (for those not having children prior to the infertility) and secondary infertility. It was associated with several medical problems arising from both the men and women. According to previous study, the most common causes of infertility are sperm abnormalities, ovulation dysfunction, and tubal pathology. However, a few of the cases remain unexplained [1]. In our case, the patient and her husband experienced primary infertility, which pushed them to try to retain the abdominal pregnancy as they were unsure if the patient could have any other pregnancy.

Abdominal pregnancy is a rare event but is associated with significant morbidity and mortality [6]. The incidence varies widely with geographical location, degree of antenatal attendance, level of medical care, and socioeconomic status. It is believed that abdominal pregnancy is more common in developing countries, probably because of the high frequency of pelvic inflammatory disease in these areas [7]. Besides, it may be due to low socioeconomic status, history of infertility, tubal sterilization, tubal reconstruction surgery, and pregnancy with intrauterine device [7].

Abdominal pregnancy is also associated with maternal mortality rate of 0.5–18% [7]. The maternal mortality rate was 7.7 times higher than that observed in tubal ectopic pregnancies and 90 times higher than in an intrauterine pregnancy [7]. Several studies considered that expectant management is possible, while others proposed that there is a high risk for a life-threatening hemorrhage [8, 9]. Therefore, early surgical termination is advised to all abdominal pregnancy patients [5]. However, there were cases of expectant management for abdominal pregnancies with satisfactory results [5, 10]. Although there were cases of successful expectant management for abdominal pregnancies, it is usually reserved for special populations, one of which is previously infertile couples [10]. Our patient in this case also experienced primary infertility prior to this pregnancy. However, the neonate died shortly after birth in this study. Therefore, we recommend that abdominal pregnancy be diagnosed and preferably terminated early to prevent further complications.

### 3.2. Psychologic Aspect

Infertility has devastating consequences to a couple psychology. Due to infertility involving an inability to achieve the desired social role as “parents,” infertility is associated with severe psychological distress for a couple [11]. This problem creates stress for the couple, increasing the risk of developing anger, depression, anxiety, and various marital problems [12]. Ironically, the psychological problems associated with infertility often increase sexual dysfunction and social isolation [12].

Along with the stress associated with the infertility itself, there is considerable stress associated with those undertaking in vitro fertilization (IVF) or other forms of infertility treatment. A previous study mentioned that the clinical depression rates of women trying to conceive using IVF are similar to those having heart disease or even cancer [13]. Therefore, psychologic assessment and guidance should be delivered to all infertile patients.

Abdominal pregnancy in our case also added additional psychologic burden to our patient and her husband. After three years of waiting, they had to accept the fact that their baby might not survive and the patient had to shoulder the additional risks associated with abdominal pregnancy.

### 3.3. Social Aspect

Infertility remains as a social problem worldwide. Due to the traditions of some countries, infertility is associated with a social stigma of being “imperfect,” especially for the women [9]. It is caused by patriarchy developed in many countries. Although recent medical advances have shown that there are as many cases of which male factor is the main factor of infertility, the societies continue to blame women as the “perpetrator” of infertility. Moreover, this phenomenon is often accompanied with the normalization of polygamy practice due to infertility [13].

There are many patients not receiving the recommended medical care according to several guidelines [14]. The suboptimal management may be caused by several factors, namely, the reluctance of the patients, the cost of infertility treatment, and the availability of proper medical infrastructure [15]. In our case, the patient and her husband had not taken any steps to address their infertility problem due to the high cost and unavailability of proper medical infrastructure in their city. Moreover, her husband worked as a merchant at a traditional market, thus accompanying her wife to other city meant no income for their family.

On the other hand, in abdominal pregnancy, we try to joint with the stockholder in their around home to help if there is any emergency situation and ready to bring her to the RSCM Hospital.

## 4. Conclusion

Abdominal pregnancy is a rare and highly morbid form of ectopic pregnancy. It demands skilled approach in management. Early diagnosis and termination are preferable to ensure the best outcome for the mother. Comprehensive and holistic approach is required in order to deliver the best outcome for the patient and her family.

## Data Availability

The data used to support this study are available from the corresponding author upon request.

## References

[1] Kamel R. M. (2010). Management of the infertile couple an evidence based protocol. *Reproductive Biology and Endocrinology*.

[2] Yasumoto K., Sato Y., Ueda Y. (2017). Expectant management for abdominal pregnancy. *Gynecology and Minimally Invasive Therapy*.

[3] Yoder N., Tal R., Martin J. R. (2016). Abdominal ectopic pregnancy after in vitro fertilization and single embryo transfer: a case report and systematic review. *Reproductive Biology and Endocrinology*.

[4] Silva P., Vargas P., Munoz A. (2018). Expectant management of an abdominal pregnancy diagnosed at 18 weeks: a case report. *Obstetrics and Gynecology International Journal*.

[5] Rohilla M., Joshi B., Jain V., Neetimala G. S., Gainder S. (2018). Advanced abdominal pregnancy: a search for consensus. Review of literature along with case report. *Archives of Gynecology and Obstetrics*.

[6] Tucker K., Bhardwaj N. R., Clark E., Espey E. (2017). Delayed diagnosis and management of second trimester abdominal pregnancy. *BMJ Case Reports*.

[7] Hailu F. G., Yihunie G. T., Essa A. A., Tsega W. K. (2017). Advanced abdominal pregnancy, with live fetus and severe preeclampsia: case report. *BMC Pregnancy and Childbirth*.

[8] Hajji A., Toumi D., Laakom O., Cherif O., Faleh R. (2020). Early primary abdominal pregnancy: diagnosis and management; A case report. *International Journal of Surgery Case Reports*.

[9] Singh Y., Singh S. K., Ganguly M., Singh S., Kumar P. (2016). Secondary abdominal pregnancy. *Medical Journal Armed Forces India*.

[10] King M., Bewes P. C., Cairns J., Thornton J. (2014). Abdominal pregnancy in primary surgery. https://www.scirp.org/(S(i43dyn45teexjx455qlt3d2q))/reference/referencespapers.aspx?referenceid=1316622.

[11] Agarwal N., Odejinmi F. (2014). Early abdominal ectopic pregnancy: challenges, update and review of current management. *The Obstetrician and Gynaecologist*.

[12] Gidiri M. F., Kanyenze M. (2015). Advanced abdominal ectopic pregnancy: lessons from three cases from Zimbabwe and a literature appraisal of diagnostic and management challenges. *Women’s Health*.

[13] Poole A., Haas D., Magann E. F. (2012). Early abdominal ectopic pregnancies a systematic review of the literature. *Gynecologic and Obstetric Investigation*.

[14] Greil A. L., Slauson-Blevins K., McQuillan J. (2010). The experience of infertility a review of recent literature. *Sociology of Health and Illness*.

[15] Norris A. S. (1965). Psychological aspects of infertility. *Journal of the Iowa Medical Society*.

